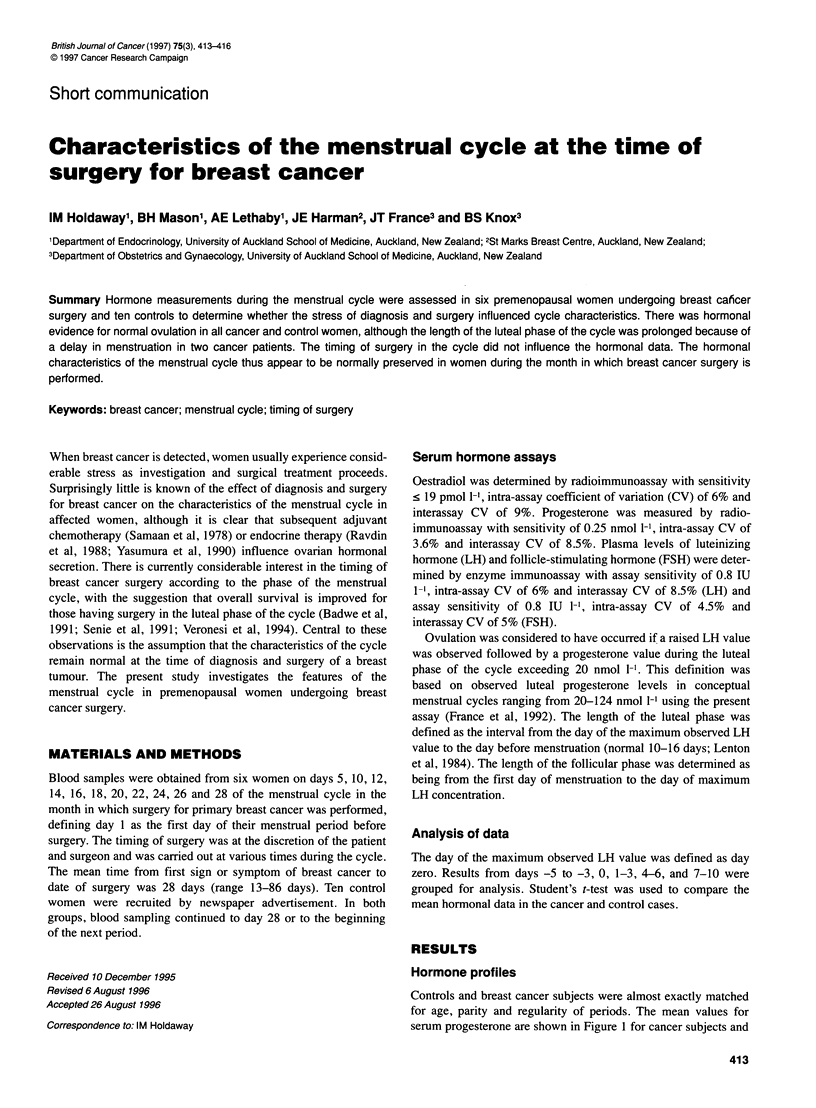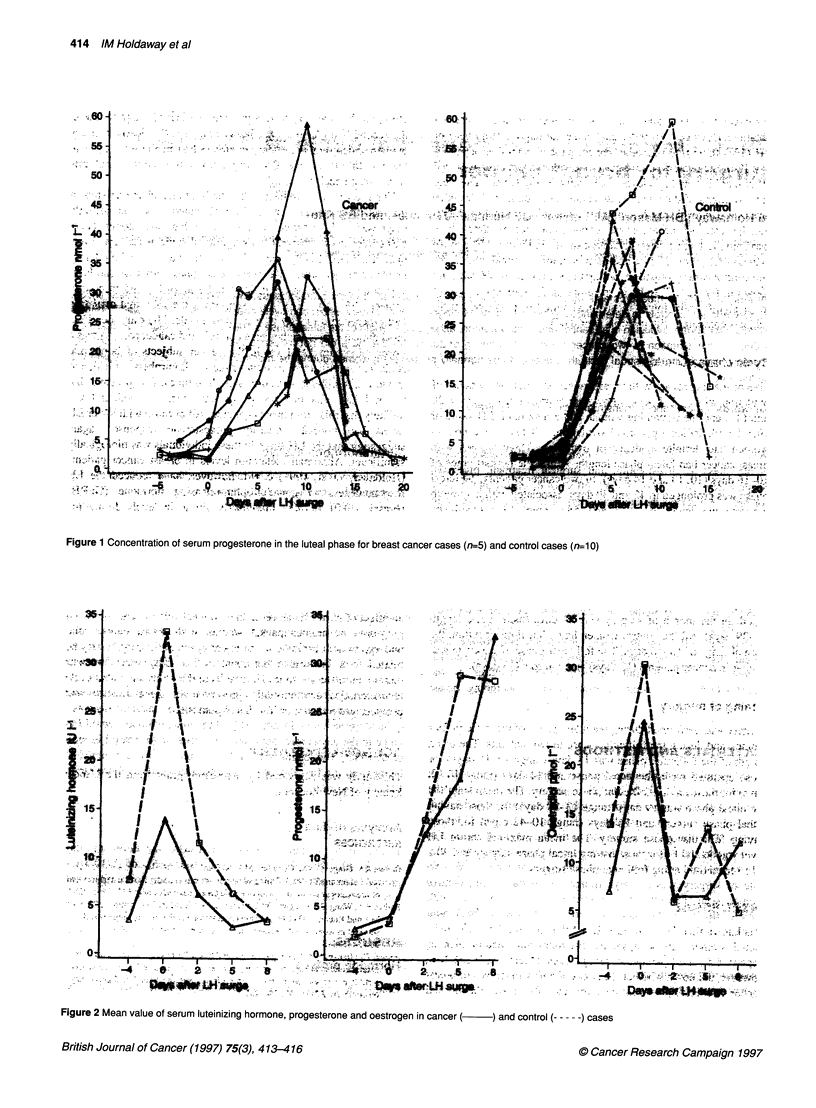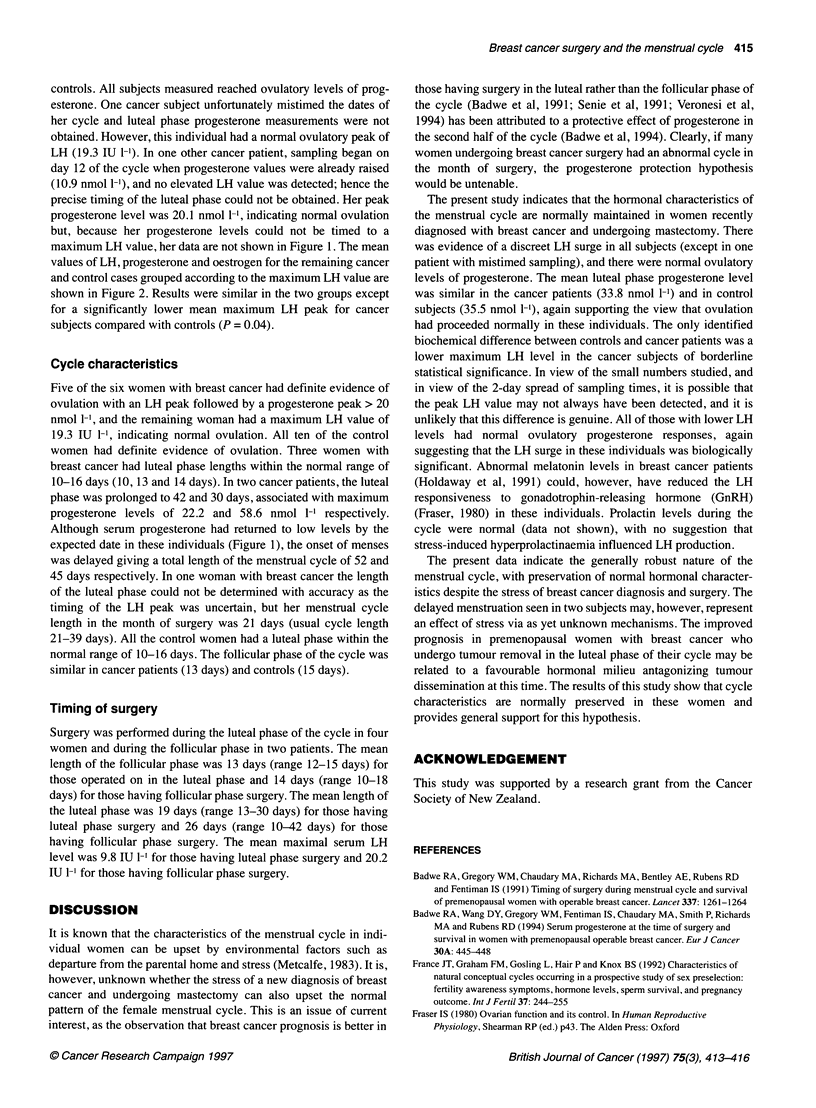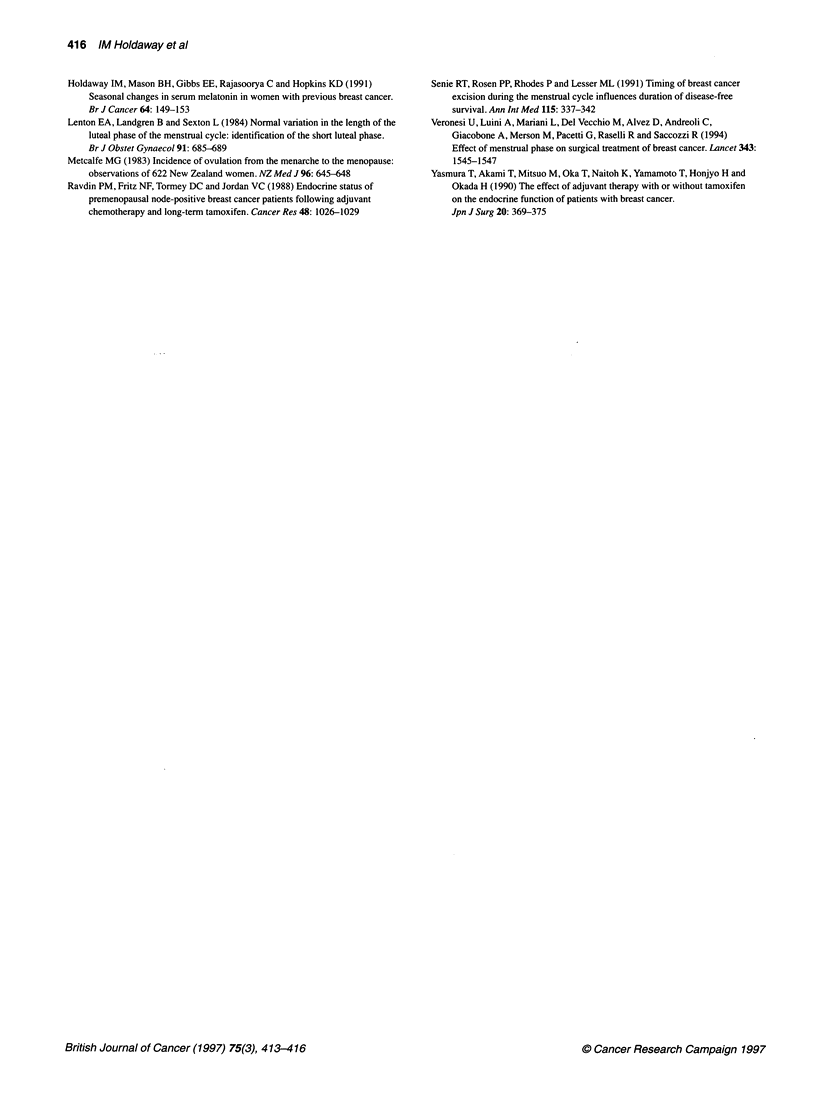# Characteristics of the menstrual cycle at the time of surgery for breast cancer.

**DOI:** 10.1038/bjc.1997.67

**Published:** 1997

**Authors:** I. M. Holdaway, B. H. Mason, A. E. Lethaby, J. E. Harman, J. T. France, B. S. Knox

**Affiliations:** Department of Endocrinology, University of Auckland School of Medicine, New Zealand.

## Abstract

Hormone measurements during the menstrual cycle were assessed in six premenopausal women undergoing breast cancer surgery and ten controls to determine whether the stress of diagnosis and surgery influenced cycle characteristics. There was hormonal evidence for normal ovulation in all cancer and control women, although the length of the luteal phase of the cycle was prolonged because of a delay in menstruation in two cancer patients. The timing of surgery in the cycle did not influence the hormonal data. The hormonal characteristics of the menstrual cycle thus appear to be normally preserved in women during the month in which breast cancer surgery is performed.


British Journal of Cancer (1997) 75(3), 413–416
© 1997 Cancer Research Campaign

Short communication

### Characteristics of the menstrual cycle at the time of surgery for breast cancer

**IM Holdaway[1], BH Mason[1], AE Lethaby[1], JE Harman[2], JT France[3] and BS Knox[3]**

[1]Department of Endocrinology, University of Auckland School of Medicine, Auckland, New Zealand; [2]St Marks Breast Centre, Auckland, New Zealand; [3]Department of Obstetrics and Gynaecology, University of Auckland School of Medicine, Auckland, New Zealand

**Summary** Hormone measurements during the menstrual cycle were assessed in six premenopausal women undergoing breast cancer surgery and ten controls to determine whether the stress of diagnosis and surgery influenced cycle characteristics. There was hormonal evidence for normal ovulation in all cancer and control women, although the length of the luteal phase of the cycle was prolonged because of a delay in menstruation in two cancer patients. The timing of surgery in the cycle did not influence the hormonal data. The hormonal characteristics of the menstrual cycle thus appear to be normally preserved in women during the month in which breast cancer surgery is performed.

**Keywords:** breast cancer; menstrual cycle; timing of surgery

When breast cancer is detected, women usually experience considerable stress as investigation and surgical treatment proceeds. Surprisingly little is known of the effect of diagnosis and surgery for breast cancer on the characteristics of the menstrual cycle in affected women, although it is clear that subsequent adjuvant chemotherapy (Samaan et al, 1978) or endocrine therapy (Ravdin et al, 1988; Yasumura et al, 1990) influence ovarian hormonal secretion. There is currently considerable interest in the timing of breast cancer surgery according to the phase of the menstrual cycle, with the suggestion that overall survival is improved for those having surgery in the luteal phase of the cycle (Badwe et al, 1991; Senie et al, 1991; Veronesi et al, 1994). Central to these observations is the assumption that the characteristics of the cycle remain normal at the time of diagnosis and surgery of a breast tumour. The present study investigates the features of the menstrual cycle in premenopausal women undergoing breast cancer surgery.

### MATERIALS AND METHODS

Blood samples were obtained from six women on days 5, 10, 12, 14, 16, 18, 20, 22, 24, 26 and 28 of the menstrual cycle in the month in which surgery for primary breast cancer was performed, defining day 1 as the first day of their menstrual period before surgery. The timing of surgery was at the discretion of the patient and surgeon and was carried out at various times during the cycle. The mean time from first sign or symptom of breast cancer to date of surgery was 28 days (range 13–86 days). Ten control women were recruited by newspaper advertisement. In both groups, blood sampling continued to day 28 or to the beginning of the next period.

*Received 10 December 1995*
*Revised 6 August 1996*
*Accepted 26 August 1996*

*Correspondence to:* IM Holdaway

### Serum hormone assays

Oestradiol was determined by radioimmunoassay with sensitivity ≤ 19 pmol $l^{-1}$, intra-assay coefficient of variation (CV) of 6% and interassay CV of 9%. Progesterone was measured by radioimmunoassay with sensitivity of 0.25 nmol $l^{-1}$, intra-assay CV of 3.6% and interassay CV of 8.5%. Plasma levels of luteinizing hormone (LH) and follicle-stimulating hormone (FSH) were determined by enzyme immunoassay with assay sensitivity of 0.8 IU $l^{-1}$, intra-assay CV of 6% and interassay CV of 8.5% (LH) and assay sensitivity of 0.8 IU $l^{-1}$, intra-assay CV of 4.5% and interassay CV of 5% (FSH).

Ovulation was considered to have occurred if a raised LH value was observed followed by a progesterone value during the luteal phase of the cycle exceeding 20 nmol $l^{-1}$. This definition was based on observed luteal progesterone levels in conceptual menstrual cycles ranging from 20–124 nmol $l^{-1}$ using the present assay (France et al, 1992). The length of the luteal phase was defined as the interval from the day of the maximum observed LH value to the day before menstruation (normal 10–16 days; Lenton et al, 1984). The length of the follicular phase was determined as being from the first day of menstruation to the day of maximum LH concentration.

### Analysis of data

The day of the maximum observed LH value was defined as day zero. Results from days –5 to –3, 0, 1–3, 4–6, and 7–10 were grouped for analysis. Student's *t*-test was used to compare the mean hormonal data in the cancer and control cases.

### RESULTS

### Hormone profiles

Controls and breast cancer subjects were almost exactly matched for age, parity and regularity of periods. The mean values for serum progesterone are shown in Figure 1 for cancer subjects and

**Figure 1** Concentration of serum progesterone in the luteal phase for breast cancer cases ($n$=5) and control cases ($n$=10)

**Figure 2** Mean value of serum luteinizing hormone, progesterone and oestrogen in cancer (———) and control (- - - - -) cases

© Cancer Research Campaign 1997

controls. All subjects measured reached ovulatory levels of progesterone. One cancer subject unfortunately mistimed the dates of her cycle and luteal phase progesterone measurements were not obtained. However, this individual had a normal ovulatory peak of LH (19.3 IU $l^{-1}$). In one other cancer patient, sampling began on day 12 of the cycle when progesterone values were already raised (10.9 nmol $l^{-1}$), and no elevated LH value was detected; hence the precise timing of the luteal phase could not be obtained. Her peak progesterone level was 20.1 nmol $l^{-1}$, indicating normal ovulation but, because her progesterone levels could not be timed to a maximum LH value, her data are not shown in Figure 1. The mean values of LH, progesterone and oestrogen for the remaining cancer and control cases grouped according to the maximum LH value are shown in Figure 2. Results were similar in the two groups except for a significantly lower mean maximum LH peak for cancer subjects compared with controls ($P = 0.04$).

### Cycle characteristics

Five of the six women with breast cancer had definite evidence of ovulation with an LH peak followed by a progesterone peak > 20 nmol $l^{-1}$, and the remaining woman had a maximum LH value of 19.3 IU $l^{-1}$, indicating normal ovulation. All ten of the control women had definite evidence of ovulation. Three women with breast cancer had luteal phase lengths within the normal range of 10–16 days (10, 13 and 14 days). In two cancer patients, the luteal phase was prolonged to 42 and 30 days, associated with maximum progesterone levels of 22.2 and 58.6 nmol $l^{-1}$ respectively. Although serum progesterone had returned to low levels by the expected date in these individuals (Figure 1), the onset of menses was delayed giving a total length of the menstrual cycle of 52 and 45 days respectively. In one woman with breast cancer the length of the luteal phase could not be determined with accuracy as the timing of the LH peak was uncertain, but her menstrual cycle length in the month of surgery was 21 days (usual cycle length 21–39 days). All the control women had a luteal phase within the normal range of 10–16 days. The follicular phase of the cycle was similar in cancer patients (13 days) and controls (15 days).

### Timing of surgery

Surgery was performed during the luteal phase of the cycle in four women and during the follicular phase in two patients. The mean length of the follicular phase was 13 days (range 12–15 days) for those operated on in the luteal phase and 14 days (range 10–18 days) for those having follicular phase surgery. The mean length of the luteal phase was 19 days (range 13–30 days) for those having luteal phase surgery and 26 days (range 10–42 days) for those having follicular phase surgery. The mean maximal serum LH level was 9.8 IU $l^{-1}$ for those having luteal phase surgery and 20.2 IU $l^{-1}$ for those having follicular phase surgery.

### DISCUSSION

It is known that the characteristics of the menstrual cycle in individual women can be upset by environmental factors such as departure from the parental home and stress (Metcalfe, 1983). It is, however, unknown whether the stress of a new diagnosis of breast cancer and undergoing mastectomy can also upset the normal pattern of the female menstrual cycle. This is an issue of current interest, as the observation that breast cancer prognosis is better in those having surgery in the luteal rather than the follicular phase of the cycle (Badwe et al, 1991; Senie et al, 1991; Veronesi et al, 1994) has been attributed to a protective effect of progesterone in the second half of the cycle (Badwe et al, 1994). Clearly, if many women undergoing breast cancer surgery had an abnormal cycle in the month of surgery, the progesterone protection hypothesis would be untenable.

The present study indicates that the hormonal characteristics of the menstrual cycle are normally maintained in women recently diagnosed with breast cancer and undergoing mastectomy. There was evidence of a discreet LH surge in all subjects (except in one patient with mistimed sampling), and there were normal ovulatory levels of progesterone. The mean luteal phase progesterone level was similar in the cancer patients (33.8 nmol $l^{-1}$) and in control subjects (35.5 nmol $l^{-1}$), again supporting the view that ovulation had proceeded normally in these individuals. The only identified biochemical difference between controls and cancer patients was a lower maximum LH level in the cancer subjects of borderline statistical significance. In view of the small numbers studied, and in view of the 2-day spread of sampling times, it is possible that the peak LH value may not always have been detected, and it is unlikely that this difference is genuine. All of those with lower LH levels had normal ovulatory progesterone responses, again suggesting that the LH surge in these individuals was biologically significant. Abnormal melatonin levels in breast cancer patients (Holdaway et al, 1991) could, however, have reduced the LH responsiveness to gonadotrophin-releasing hormone (GnRH) (Fraser, 1980) in these individuals. Prolactin levels during the cycle were normal (data not shown), with no suggestion that stress-induced hyperprolactinaemia influenced LH production.

The present data indicate the generally robust nature of the menstrual cycle, with preservation of normal hormonal characteristics despite the stress of breast cancer diagnosis and surgery. The delayed menstruation seen in two subjects may, however, represent an effect of stress via as yet unknown mechanisms. The improved prognosis in premenopausal women with breast cancer who undergo tumour removal in the luteal phase of their cycle may be related to a favourable hormonal milieu antagonizing tumour dissemination at this time. The results of this study show that cycle characteristics are normally preserved in these women and provides general support for this hypothesis.

### ACKNOWLEDGEMENT

This study was supported by a research grant from the Cancer Society of New Zealand.

### REFERENCES

Badwe RA, Gregory WM, Chaudary MA, Richards MA, Bentley AE, Rubens RD and Fentiman IS (1991) Timing of surgery during menstrual cycle and survival of premenopausal women with operable breast cancer. *Lancet* **337**: 1261–1264

Badwe RA, Wang DY, Gregory WM, Fentiman IS, Chaudary MA, Smith P, Richards MA and Rubens RD (1994) Serum progesterone at the time of surgery and survival in women with premenopausal operable breast cancer. *Eur J Cancer* **30A**: 445–448

France JT, Graham FM, Gosling L, Hair P and Knox BS (1992) Characteristics of natural conceptual cycles occurring in a prospective study of sex preselection: fertility awareness symptoms, hormone levels, sperm survival, and pregnancy outcome. *Int J Fertil* **37**: 244–255

Fraser IS (1980) Ovarian function and its control. In *Human Reproductive Physiology*, Shearman RP (ed.) p43. The Alden Press: Oxford

© Cancer Research Campaign 1997

Holdaway IM, Mason BH, Gibbs EE, Rajasoorya C and Hopkins KD (1991) Seasonal changes in serum melatonin in women with previous breast cancer. *Br J Cancer* **64**: 149–153

Lenton EA, Landgren B and Sexton L (1984) Normal variation in the length of the luteal phase of the menstrual cycle: identification of the short luteal phase. *Br J Obstet Gynaecol* **91**: 685–689

Metcalfe MG (1983) Incidence of ovulation from the menarche to the menopause: observations of 622 New Zealand women. *NZ Med J* **96**: 645–648

Ravdin PM, Fritz NF, Tormey DC and Jordan VC (1988) Endocrine status of premenopausal node-positive breast cancer patients following adjuvant chemotherapy and long-term tamoxifen. *Cancer Res* **48**: 1026–1029

Senie RT, Rosen PP, Rhodes P and Lesser ML (1991) Timing of breast cancer excision during the menstrual cycle influences duration of disease-free survival. *Ann Int Med* **115**: 337–342

Veronesi U, Luini A, Mariani L, Del Vecchio M, Alvez D, Andreoli C, Giacobone A, Merson M, Pacetti G, Raselli R and Saccozzi R (1994) Effect of menstrual phase on surgical treatment of breast cancer. *Lancet* **343**: 1545–1547

Yasmura T, Akami T, Mitsuo M, Oka T, Naitoh K, Yamamoto T, Honjyo H and Okada H (1990) The effect of adjuvant therapy with or without tamoxifen on the endocrine function of patients with breast cancer. *Jpn J Surg* **20**: 369–375

© Cancer Research Campaign 1997